# Roles of Multiple Flagellins in Flagellar Formation and Flagellar Growth Post Bdelloplast Lysis in *Bdellovibrio bacteriovorus*

**DOI:** 10.1016/j.jmb.2009.10.003

**Published:** 2009-12-18

**Authors:** Yoshiko Iida, Laura Hobley, Carey Lambert, Andrew K. Fenton, R. Elizabeth Sockett, Shin-Ichi Aizawa

**Affiliations:** 1 Department of Life Sciences, Prefectural University of Hiroshima, 562 Nanatsuka, Shobara, Hiroshima 727-0023, Japan; 2 Institute of Genetics, School of Biology, University of Nottingham, Queens Medical Centre, Nottingham NG7 2UH, UK

**Keywords:** EM, electron microscopy, RT, reverse transcription, *Bdellovibrio bacteriovorus*, flagellin, tapered wave, 2D gel, polymorphism flagellin secretion

## Abstract

*Bdellovibrio bacteriovorus* cells have a single polar flagellum whose helical pitch and diameter characteristically change near the midpoint, resulting in a tapered wave. There are six flagellin genes in the genome: *fliC1* to *fliC6*. Accordingly, the flagellar filament is composed of several similar flagellin species. We have used knockout mutants of each gene and analyzed the mutational effects on the filament length and on the composition and localization of each flagellin species in the filament by electron microscopy and one- and two-dimensional polyacrylamide gel electrophoresis. The location and amounts of flagellins in a filament were determined to be as follows: a small amount of FliC3 at the proximal end, followed by a large amount of FliC5, a large amount of FliC1, a small amount of FliC2 in this order, and a large amount of FliC6 at the distal end. FliC4 was present at a low level, but the location was not determined. Filament lengths of newly born progeny cells increased during prolonged incubation in nutrient-deficient buffer. The newly formed part of the elongated filament was composed of mainly FliC6. Reverse transcription PCR analysis of flagellar gene expression over 5 days in buffer showed that *fliC* gene expression tailed off over 5 days in the wild-type cells, but in the *fliC5* mutant, expression of the *fliC2*, *fliC4*, and *fliC6* genes was elevated on day 5, suggesting that they may be expressed to compensate for the absence of a major component, FliC5.

## Introduction

*Bdellovibrio bacteriovorus* is a small predatory Gram-negative bacterium that lives by predation on other Gram-negative bacteria.^1^ The *B. bacteriovorus* cell has a single, polar, sheathed flagellum and is actively motile to forage for prey cells.^2^ The flagellum appears unique in its shape as shown by electron microscopy (EM); it has typically three or four waves with smaller waves toward the distal end, thus appearing as a tapered wavy filament.^3,4^

The shape of a flagellar filament is helical and uniquely determined by three geometrical parameters: pitch, helical diameter, and handedness.^5^ There are three families of flagella defined by distinctive helical parameters: family I includes peritrichous flagella with large pitches and diameters, family II includes polar flagella with medium pitches and diameters, and family III contains lateral flagella with small pitches and diameters.^6^ There are exceptions that do not belong to these three families because their flagella have an irregular shape. Interestingly, the exceptional flagella are mostly produced by alpha-proteobacteria.^6^ Although *B. bacteriovorus* are in the delta-proteobacteria, its flagella belong to this irregularly shaped group of flagella.

Flagellar genes of *B. bacteriovorus* are scattered all over the genome, forming small clusters of two or three genes,^7^ similar to those of *Helicobacter pylori* or *Caulobacter crescentus*. Interestingly, all these three species have multiple flagellin genes in their genome, six (*fliC1*–*fliC6*) in *B. bacteriovorus*,^7^ two (*flaA* and *flaB*) in *H. pylori*,^8^ and five (*fljK*–*fljO*) in *C. crescentus*.^9^ It also should be noted that the flagella of *B*. *bacteriovorus* and *H*. *pylori* are both sheathed with a membranous material continuous with the outer membrane. In *C. crescentus*, each flagellin was identified to localize as a block in a filament.^9^ However, the role of each flagellin species for the filament was not clear.

In this study, we used flagellin knockout mutants to identify the location of each flagellin species and its role in the *Bdellovibrio* flagellar filament. Filaments were purified by cesium chloride (CsCl) density gradient ultracentrifugation, and flagellins were separated by one-dimensional (1D) and 2D gel electrophoresis. Reverse transcription PCR (RT-PCR) was also employed to observe the expression of flagellin genes. The role of each flagellin in filament formation is discussed.

## Results

### Six flagellin genes in the *Bdellovibrio* genome

**Fig. 1:**
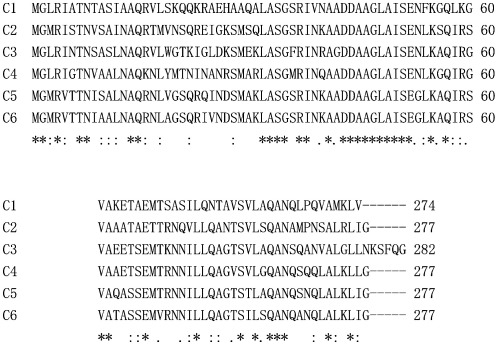
Alignment of terminal regions of *B. bacteriovorus* multiple flagellins. The first 60 and last 40 amino acids of each flagellin were aligned. Identical amino acids are marked by stars.

There are six flagellin genes scattered in the genome of *B. bacteriovorus* HD100 strain, and these are conserved in 109J strain used in this study: a pair of the *fliC1* and *fliC2* genes with the locus tags Bd0604 and Bd0606 (in the whole genome size of 3780 kb), a pair of the *fliC3* and *fliC4* genes with Bd0408 and Bd0410, the *fliC5* gene with Bd3052, and the *fliC6* gene with Bd3342. ^4,7^ In general, amino acid sequences of the terminal regions of flagellin molecules are highly conserved, and this is true for *B. bacteriovorus* flagellins; the first 60 and last 40 amino acid sequences of the flagellins were highly homologous (Fig. 1). Accordingly, the molecular sizes and isoelectric points (p*I*s) of those flagellins are very similar with each other; the sequence identity of each flagellin against FliC2 (which gave the highest scores) was more than 55% (Table 1).

In order to identify the location and role of each flagellin species in the filament, we used existing knockout mutants of each flagellin gene: *fliC1*–*fliC6* mutants.^4^ The behaviour of those mutant cells was observed by dark-field and phase-contrast microscopy; filament structures were analyzed by EM; and their components were determined by 1D and 2D SDS–polyacrylamide gel electrophoresis (SDS-PAGE).

### Behaviour and morphology of flagellin deletion mutants

**Fig. 2:**
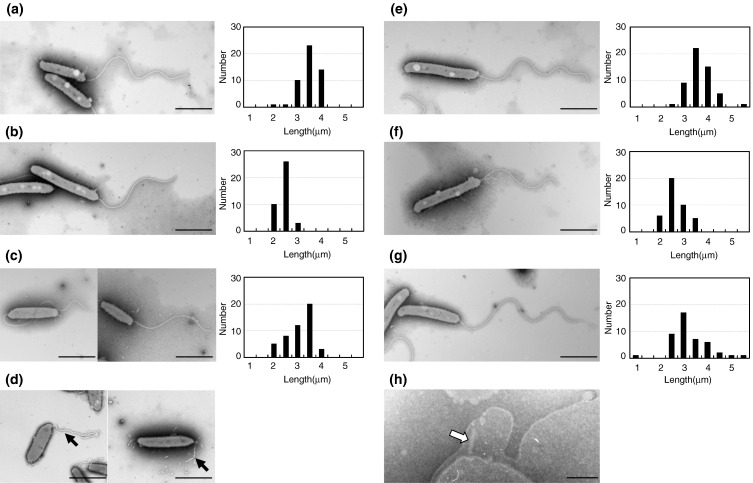

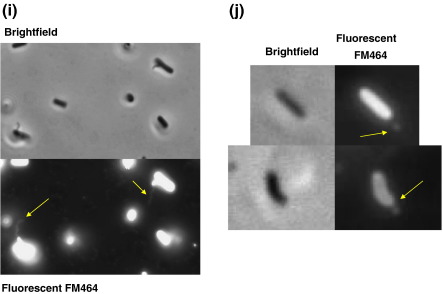
EM images of flagella from flagellin knockout mutants: (a) wild-type 109J, (b) *fliC1*, (c) *fliC2*, (d) *fliC3*, (e) *fliC4*, (f) *fliC5*, and (g) *fliC6* mutants and (h) a hook–basal body complex in the *fliC3* mutant. Bars represent 1 μm except for (h) which is 100 nm. Histograms showing the distribution of flagellar lengths at 24 h of incubation for each strain are shown to the right of EM images. Fluorescent microscope images of (i) *Bdellovibrio* and *E. coli* RP437 (both highly motile in this culture) mixed together and stained with FM464 membrane stain. Only the *Bdellovibrio* (which are attached to the *E. coli*, preying on them) show flagellar staining, despite the *E. coli* having many non-sheathed flagella that do not stain. (j) *B. bacteriovorus* 109J *fliC3* mutant cells stained with the FM464, showing disordered membrane bleb at the pole of the cell.

The *fliC3* knockout mutant was immotile and did not have flagellar filaments (we call them just filaments hereinafter), although it produced a flagellar sheath (see below). In contrast, all other flagellin mutants were flagellated and motile,^4^ indicating that each flagellin is dispensable for other flagellins to form a filament. To elucidate the location of each flagellin in the filament, the length and shape of the filaments of each flagellate mutant were compared with those of the wild-type strain 109J by EM (Fig. 2a). The average length of the wild-type filaments was 3.8 μm with a standard deviation of 0.4 μm (sampling number *n* = 49).

(1) *FliC1*. The filament from the *fliC1* mutant looked regularly curved throughout the length without a tapered end (Fig. 2b). The distal tapered part was too short to recognize in 24 h of incubation (2.7 ± 0.3 μm, *n* = 39) (Fig. 2b, right) but distinguishably elongated after 3 days. Thus, it will be reasonable to locate FliC1 at the junction between the regular-shaped proximal half and the tapered distal half. Without FliC1, initiation of the distal part of the filament may be delayed (see next).
(2) *FliC2*. The filament length of the *fliC2* mutant was shorter than that of the wild type (Fig. 2c). In a previous paper,^4^ we reported that the filament length of the *fliC2* mutant was the same as that of the wild type. In fact, filament length is sensitively dependent on incubation time, suggesting the possibility that kinetics of filament growth may be different between mutants and the wild type (see sections below). In 24 h of incubation, the average length of the *fliC2* filaments was 3.4± 0.6 μm (*n* = 48) (Fig. 2c, right), but a distribution of both long and short filaments gave this mean value. We also noticed that the proximal portion of the filament often looked more curved and bent back around the cell body (Fig. 2c, left). This may be related to the swimming behaviour of the *fliC2* mutant, which often backs up in a manner that we have not observed in other mutants (data not shown). If the flagellar motor rotates in the reverse direction, the *fliC2* mutant filament may bend back, due to its shortness, to push rather than to pull the cell body.
(3) *FliC5*. The filament length of the *fliC5* mutant was shorter than that of the wild type, 2.9 ± 0.4 μm (*n* = 41) (Fig. 2f, right). In the *fliC5* mutant, the regular-shaped proximal part, but not the tapered distal part, of the filament is shorter than that of the wild type (Fig. 5, a *versus* b, Day 1). Thus, we suggest that FliC5 locates proximal to FliC1.
(4) *FliC4*. Both filament length and shape of the *fliC4* mutant were indistinguishable from those of the wild type; 3.9 ± 0.5 μm (*n* = 53) (Fig. 2e).
(5) *FliC6*. The filaments of the *fliC6* mutant were on average slightly shorter than those of the wild type (3.5 ± 0.8 μm, *n* = 44) (Fig. 2g) but showed diverse length distributions of both longer and shorter than those of the wild type (right, Fig. 2g). During prolonged incubation, the filament did not elongate as much as the other mutant filaments.
(6) *FliC3*. Although the *fliC3* mutant does not have filaments, it still has the filament sheath, which looked devoid of an intact filament when observed by EM (Fig. 2d). The mutant also should have the flagellar basal structure, and indeed, we found the basal structure formed up to the hook at a pole of the cells, which was visualized within the membrane in an osmotically shocked cell (Fig. 2h).^10^ Therefore, FliC3 is required at the beginning of filament formation after the hook and HAP region are formed, and the other flagellins cannot polymerize into a filament without FliC3.

That a membranous flagellar sheath was present on wild-type *Bdellovibrio* flagella was also confirmed by staining highly motile *B. bacteriovorus* wild type, mixed with highly motile *Escherichia coli* RP437 wild type, with FM464 membrane stain from Invitrogen (Fig. 2i). Only the *Bdellovibrio* showed stained flagella, despite the *E. coli* having bundles of flagella, all of which are known to be unsheathed. That the sheath formed independently of the flagellar filament was confirmed in the *fliC3* mutant by Fm464 staining of empty sheaths that are seen in this strain (Fig. 2j).^4^

### Analysis of flagellin components in the filament

**Fig. 3:**
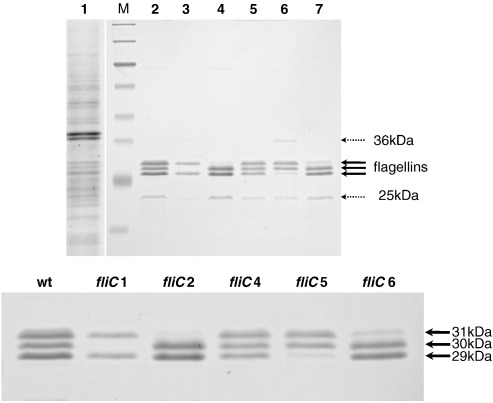
One-dimensional SDS gels of flagellin bands from isolated filaments. Lane 1, crude filaments; M, molecular markers; lane 2, purified filaments from wild-type 109J; lane 3, *fliC1*; lane 4, *fliC2*; lane 5, *fliC4*; lane 6, *fliC5*; lane 7, *fliC6* mutants. Thick arrows indicate flagellins, and thin arrows (25 and 36 kDa) indicate contaminants. Lower panels, enlarged images of flagellin bands from upper panel. Three major bands were marked as 29, 30, 31 kDa according to their molecular sizes.

**Fig. 4:**
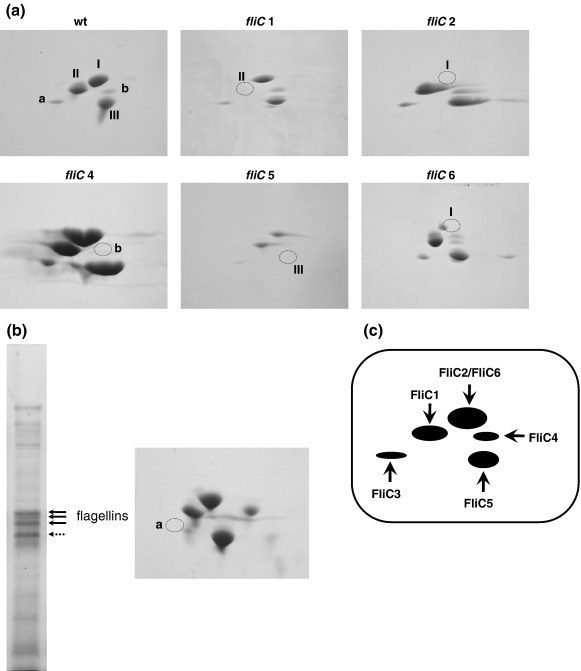
(a) Two-dimensional gels of flagellins in filament purified from wild-type 109J, *fliC1*, *fliC2*, *fliC4*, *fliC5*, and *fliC6* mutants. Three major spots were labeled as I, II, III, and two small spots as a and b. Missing spots in mutant preparations are marked by dotted circles. (b) 1D (left) and 2D (right) gels of flagellins recovered from spent culture media from the *fliC3* mutant. Thick arrows indicate flagellin bands and the dotted arrow indicates a contaminant. (c) Summary of 2D gel analysis of flagellins, FliC1–6.

We isolated filaments from cells by passing them through a pipette with a tapered tip. When isolated filaments were analyzed by SDS-PAGE, there appeared several adjacent bands at around 30 kDa, and many other bands in the background, probably derived from cell debris (Fig. 3, lane 1). To completely remove cell debris that contaminated the flagellin bands, we further purified filaments by CsCl density gradient ultracentrifugation (images of purified filament preparations are not shown). The filaments purified from the wild-type strain reproducibly gave rise to three dominant bands at 29, 30, and 31 kDa (Fig. 3, lane 2). A band at 25 kDa was a contaminant, judging from its molecular size and inconsistent appearance from preparation to preparation. Another band at 36 kDa was determined to be the outer membrane protein OmpF by amino acid sequencing (data not shown). Filaments were purified from all mutants except the nonflagellate *fliC3* mutant in the same way and were analyzed in SDS gels to assign FliCs to bands on SDS gels. The *fliC1* mutant filament lacked the 30-kDa band (Fig. 3, lane 3); the *fliC2* mutant lacked the 31-kDa band (lane 4); the *fliC4* mutant initially seemed to lack none (lane 5, but see later when this is resolved in Fig. 4c); the *fliC5* mutant lacked the 29-kDa band (lane 6); and the *fliC6* mutant lacked a large part of the 31-kDa band (lane 7).

Since the molecular sizes and isoelectric points of flagellins are very similar to each other (Table 1), there could be more than two protein bands overlapping in one band. In fact, FliC2 and FliC6 flagellins seemed to overlap at the 31-kDa band, judging from the difference of band density between the two (Fig. 3, lower panel). Thus, for further analysis, we separated those flagellins in electrofocusing gels, followed by SDS gels. In 2D gels, three major spots (I, II, and III) and two minor spots (a and b) were reproducibly observed in the wild-type filament (Fig. 4a). *fliC1* mutant filament lacked spot II; *fliC2* filament lacked spot I; *fliC4* filament lacked one of the minor spots (b); *fliC5* filament lacked spot III; and *fliC6* filament lacked a large part of spot I. It should be noted that a small spot that was absent in *fliC2* filament became visible in the *fliC6* filament preparation, indicating that FliC2 and FliC6 locate at the same position in different amounts. Taking account of the observation that the *fliC2* mutant produced more shorter filaments than the *fliC6* mutant (Fig. 2; c *versus* g), and assuming that there is no overexpression of one *fliC6* due to the absence of *fliC2* and *vice versa*, supported by the observation of the same intensity of protein bands/spots in mutants *versus* those in wild type (Fig. 3), we suggest that FliC2 is necessary for FliC6 to be efficiently incorporated in the filament, while FliC6 is not required for FliC2 to be in the filament. In short, FliC2 locates proximal to FliC6 in the filament.

Although the *fliC3* mutant does not produce flagellar filaments, we found that it does produce a hook–basal body complex (Fig. 2h). Thus, it may secrete other flagellins into the medium within the flagellar sheath, or beyond where it leaks. Indeed, when we collected proteins by trichloroacetic acid precipitation from the spent media of the *fliC3* mutant culture and analyzed them by SDS-PAGE, there appeared many bands of flagellins in 1D gels (Fig. 4b, left). All major spots (I, II, and III) and a minor spot (b) were detected, but the minor spot (a) was missing in 2D gels (Fig. 4b, right). Thus, we suggest that the minor spot (a) is FliC3.

Comparing the missing spots in mutant filament preparations, we identified the locations of all flagellin species in the gels. In conclusion, FliC1 and FliC5 form a single major spot II at 30 kDa and spot III at 29 kDa, respectively. FliC2 and FliC6 localized at the same major spot I at 31 kDa; FliC2 forms a small spot that is hidden under the major spot of FliC6. FliC3 forms a minor spot (a) at 29 kDa, and FliC4 the other minor spot (b) at 30 kDa (Fig. 4c).

### Effects of prolonged cultivation on filament length and flagellin ratio

**Fig. 5:**
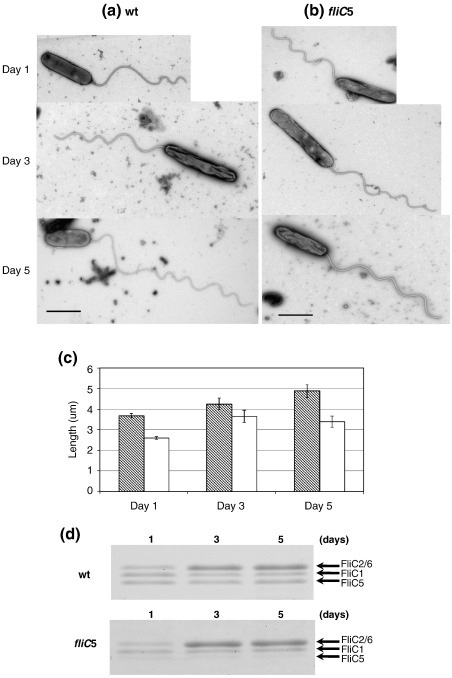
EM images of *B. bacteriovorus* wild-type strain 109J and *fliC5* mutant during starvation. (a) Wild-type cells, 24 h, 3 days, and 5 days and (b) fliC5 mutant cells, 24 h, 3 days, and 5 days after start of experiment. Cells were stained with 0.5% uranyl acetate. Scale bars represent 1 μm. (c) Average filament lengths of wild type (shaded bars) and *fliC5* mutant (empty bars) during starvation. Error bars show the 95% CI around the mean for each sample. Fifty cells of each sample were analyzed from each of two biological replicates, giving sample sizes of 100 for each data set. (d) 1D gel patterns of flagellin bands from wild type (left) and *fliC5* mutant (right).

Studies mentioned so far were routinely carried out with 1-day-old cultures of attack-phase *Bdellovibrio* cells that had emerged within 24 h from bdelloplasts of prey *E. coli* cells. During prolonged incubation in calcium Hepes buffer, after emergence from bdelloplasts, the average filament length of the *Bdellovibrio* cells was significantly elongated. This was striking, as Bdellovibrio “attack-phase cells” are not reported to take up nutrients and grow outside prey. The wild-type filament lengths on day 3 and day 5 were longer than those of day 1, and filaments on day 5 were also longer than those of day 3 (Fig. 5a and c). Since the filament length of the *fliC5* mutant was shorter than that of the wild type in 1-day culture, we measured the filament length of the *fliC5* mutant in prolonged incubations. The *fliC5* mutant was grown in calcium Hepes buffer, after release from the bdelloplast, for 3 and 5 days. Filaments were isolated, purified, and analyzed by EM and SDS-PAGE as mentioned above. By EM, the average filament length of the *fliC5* mutant was significantly longer on both day 3 and day 5 compared to day 1 (Fig. 5b). Although wild-type filaments continuously elongated for 5 days, *fliC5* filament stopped elongation on day 3; there was no significant difference in the average filament lengths between day 3 and day 5 for the *fliC5* mutant (Fig. 5c) [error bars representing 95% confidence interval (CI) for *fliC5* overlap with the mean of the other days' data sets for days 3 and 5 but not day 1; they do not overlap for any of the wild-type data].

Since the flagellar filament grows from the distal end by the addition of nascent flagellin monomers at the tip, the new portion of elongated filaments must contain more distally located flagellins than those of the 1-day culture. Using this phenomenon, we may be able to further analyze the stoichiometry of flagellin components in the filament. In 1D gels, the density of the 31-kDa band (corresponding to FliC2/6) significantly increased in the *fliC5* mutant as the incubation time passes (Fig. 5d). Since FliC6 is the major component of the 31-kDa band and locates distal to FliC2, we conclude that only FliC6 flagellin was being added to the growing filament in large quantities, in prolonged incubations. Accordingly, the filament length of the *fliC6* mutant did not elongate after 5 days, albeit slightly longer than that after 1 day (data not shown). Thus, we reconfirmed that FliC5 locates in the proximal half of the filament but is not required for FliC2/FliC6 to polymerize into filament.

### *fliC* gene expression during prolonged cultivation

We showed that wild-type filaments elongate during prolonged incubation for 5 days in buffer after release from the bdelloplast. This elongation was not as pronounced in *fliC5* mutant cells. To elucidate this filament growth under the same conditions, we carried out semiquantitative RT-PCR to observe the expression levels of flagellin genes.

**Fig. 6:**
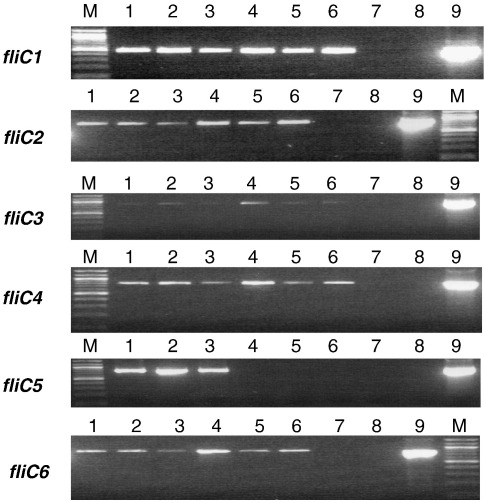
RT-PCR using FliC-specific primers and matched total RNA concentrations for *B. bacteriovorus* wild-type (WT) strain 109J and *fliC5* mutant strains cultured as attack-phase motile cells in calcium Hepes buffer for 1, 3, or 5 days after lysis of prey cells. Lanes: M, 100-bp marker (NEB); lane 1, WT day 1; lane 2, WT day 3; lane 3, WT day 5; lane 4, *fliC5* day 1; lane 5, *fliC5* day 3; lane 6, *fliC5* day 5; lane 7, *E. coli* DFB225 as a negative control; lane 8, no template; lane 9, WT genomic DNA as a positive control (25 cycles of amplification were carried out for *fliC1* and *fliC2* and 30 cycles for *fliC3* to *fliC6*).

RNA was prepared from *Bdellovibrio* attack-phase cultures, on days 1, 3, and 5 after release from bdelloplasts, along with *E. coli* as a control. For the wild-type strain the expression level of all six of the *fliC* genes appeared to stay at a nearly constant level between days 1 and 3 (Fig. 6, lanes 1 and 2). By day 5, the expression level of each gene appears to be reduced from that of day 3 (lane 3), suggesting that the maximum length of the filaments had been reached by this point (day 5) and significant expression of the flagellin genes was no longer a requirement for the cells. In contrast, in the *fliC5* mutant (Fig. 6, lanes 4 to 6), the expression levels of the *fliC2*, *fliC4*, and *fliC6* genes did not drop as much at day 5 as was seen for the wild type (Fig. 6, lanes 1 to 3; compare higher levels in lanes 6 *versus* lanes 3). This supports, albeit partially, the idea that FliC 6 and possibly FliC2 expression may be induced to compensate for the lack of FliC5, as was suggested from the 1D SDS-PAGE above (Fig. 5d). The level of FliC4 protein expression is probably too low to confirm the difference in amounts in SDS gels.

**Fig. 7:**
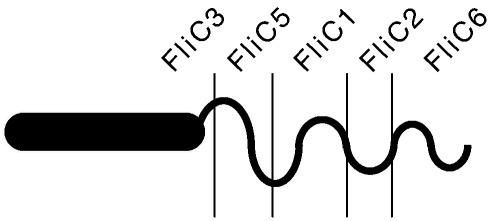
A cartoon presentation of a filament showing the relative location of each flagellin species.

In summary, all except FliC3 flagellin were dispensable for filament formation. Each flagellin species forms a block in a filament, whose location was schematically indicated in Fig. 7. FliC3 is the first flagellin essential for starting filament formation; without FliC3 no filament is formed. FliC5 is a major component needed to form the proximal part of the filament. FliC1 locates at the midpoint of the filament. FliC2 is a minor protein necessary for FliC6 to elongate efficiently; without FliC2, initiation of FliC6 filament may be delayed. In prolonged incubation in nutrient-deficient buffer, filaments were elongated by the addition of newly synthesized FliC6 at the distal end. FliC4 always appears as a minor component in the wild-type filament, but it seems dispensable because its absence did not affect the filament length.

## Discussion

### Roles of multiple flagellins

We have successfully analyzed the flagellin composition in the *B. bacteriovorus* flagellar filament. All flagellin species except FliC4 were positively identified in their position in the filament; FliC3 locates at the proximal end in the filament, followed by FliC5, FliC1, FliC2, and FliC6 at the distal end. This reminds us of multiple flagellins in *C. crescentus*.^9^ They could luckily stain each flagellin block in the filament by antibodies raised against each flagellin species. Close similarity of amino acid sequences among *Bdellovibrio* flagellins discouraged us from taking the same serological and biochemical approaches. Instead, we constructed and analyzed knockout mutants of each flagellin gene. Fortunately, some mutants showed unique filament length and shape different from those of the wild-type filament, so that we could, albeit incompletely, identify the role of those flagellin species in the filament. In the *fliC2* mutant, a more cell-proximally curved and possibly deformable flagellum was observed; as with the other single *fliC* mutants, the defect may be due to the absence of a pure filament region of a single FliC, or the alteration of the composition of a mosaic region of the filament that includes this FliC and other(s).

### Why and how is the filament tapered?

The taperedness of the *Bdellovibrio* flagellar filament was first reported by Thomashow and Rittenberg.^2^ It is not known to date whether flagella from any other bacterial species might have this kind of tapered wavy filament. It is not clear why the *Bdellovibrio* flagellar filament must have such a strange shape. For the sake of argument, we could postulate that the shape gives a hydrodynamic advantage to a cell to swim, possibly close to prey cells, or that the tapered shape is necessary for maintaining the filament at its optimal length (about 4 μm). However, since those proposals are hard to prove for the moment, we decided to stick to the analysis of the detailed structure of the filament in this study.

Lambert *et al*.^4^ suggested that the *fliC5* gene was responsible for the smaller-amplitude waveforms at the distal end of the filament, as the *fliC5* mutant had shorter filaments. Our data do not support this opinion anymore. We showed that FliC5 was a major component of the proximal half of the filament. This is confirmed by different shapes of elongated filaments between the wild type and the *fliC5* mutant (Fig. 5a and b; compare the shapes of the proximal portions). It will be interesting to know whether the lattice structures of these two filament parts are similar to or completely different from each other. However, at present there is no method to determine the lattice structure of curved filaments.

The RT-PCR expression data show that the expression of each of the *fliC* genes is being reduced for the wild-type strain by day 5 in comparison to day 3. For the *fliC5* mutant, increase in filament length is seen between day 1 and day 3, however no significant growth is seen between days 3 and 5. This correlates with the reduction in *fliC* expression in this strain on day 3 compared to day 1, suggesting that most of the flagellum lengthening occurs in the first 2 to 3 days after bdelloplast lysis for this strain.

It is interesting to note that interrupting only one of the flagellin genes, *fliC3*, results in inability to form a filament. This is in contrast to the situation in the nonpredatory *Vibrio* species where any of the six flagellins can be deleted without abolishing motility.^11^ Although we showed previously that motility is not required for prey penetration, it is clearly useful and important for encountering prey cells in liquid media.^4^ It is hard to determine why *fliC3* deletion abolishes motility, when the *Bdellovibrio* have seemingly undergone *fliC* gene duplication to prevent “easy” abolition of motility by natural *fliC* mutation. It may simply indicate that FliC3 is the only flagellin that can form a junction to the hook and hook-associated proteins, before other flagellins can be exported and added on. Or it may indicate that FliC3 expression acts naturally as a developmental checkpoint in the *Bdellovibrio* life cycle, a cycle that involves a nonmotile phase in the periplasm where *Bdellovibrio* are septating from a coenocytic filament, but where shortly the septated *Bdellovibrio* must rapidly and coordinately become motile and break free from the prey bdelloplast. It could be that synthesizing the flagella up to the *fliC3* stage, including basal body and hook and flagellar sheath (for *fliC3* mutant, cells still produce flagellar sheaths), allows for a rapid completion of a functional flagellum by induction of FliC3 expression when divided *Bdellovibrio* cells are ready to lyse the bdelloplast. Certainly, *fliC3* expression is off midway through the predatory cycle (data not shown). We do not know if any exogenous signal from the exhausted bdelloplast regulates *fliC3* expression, but this would be an expedient way of completing flagellar synthesis.

Our data here indicate that FliC3 and the other five *Bdellovibrio* flagellins are all involved in the complex construction of this unique waveform filament at different stages in the motile lifestyle. It is possible that the tapering waveform of the *Bdellovibrio* filament is hydrodynamically important to motility near and at the surface of other bacteria in wild environments, but this requires a further biophysical analysis. If this is the case, then the multiple flagellins of the *Bdellovibrio* filament exist to produce a sculpted propeller to optimize hydrodynamic forces at the prey–cell interface, rather than to provide a set of “spare-part” flagellins to build a flagellum from at all times. Because we grow and microscopically monitor the predatory efficiency of *Bdellovibrio fliC* mutants in optimized temperatures and fixed buffer compositions and predator–prey ratios, we may not see negative effects of having flagella missing a single FliC in the laboratory, but there may be a meaningful diminution in predatory efficiency in the wild. The further observation that filament length is modulated in response to time in buffer, without prey, outside the *Bdelloplast* cell is interesting. This suggests that, in the wild, the modulation of filament length, in response to starvation due to lack of prey encounters, might be important in generating the necessary speed, as Lambert *et al*. ^4^ have shown, that *Bdellovibrio* cells with shorter filaments swim, more slowly, or distance over which a *Bdellovibrio* cell can swim to allow it to locate prey before it runs out of stored energy. As this is a costly process in terms of flagellar protein synthesis, presumably from stored amino acids, it must be a last resort for a predatory bacterium.

## Materials and Methods

### Strains and growth conditions

*B. bacteriovorus* 109J and its derivatives used in this study are all from a previous paper.^4^ Cells were grown by conventional methods using *E. coli* nonflagellate strain DFB225 (pCL100) as the prey.^4,12^

### Purification of flagellar filaments

Flagellar filaments were isolated from cells by pipetting for 30–60 min and separated from cells by several low-speed centrifugations. Crude filaments were further purified by CsCl density gradient ultracentrifugation. In a density of 36% CsCl solution, the filament made a tight thin band at the middle of the tube. The band was recovered with a pipette with a tapered mouth to avoid other fractions above and below the band. Filament samples were diluted with phosphate buffer and precipitated by ultracentrifugation to remove abundant CsCl. Pellets were resuspended in phosphate buffer and used for further experiments.

### Microscopy

For visualization by EM, cells were washed with distilled water by centrifugation at low speeds (2000–3000 rpm) to prevent cell burst. Samples were stained with 1% phosphotungstic acid (pH 7) or 0.5% uranyl acetate (pH 4) and observed at 80 kV with either a JEOL 1200EX (Japan) or a JEOL JEM1010 (UK) electron microscope.

### Measurements of filament length

Filament length was measured to the nearest 0.01 μm on each of 100 cells for each strain. *Bdellovibrio* were again grown in predatory cultures with DFB225 (pCL100) for 24 h before being sampled and stained with 1% phosphotungstic acid (pH 7.0) or 0.5% uranyl acetate (pH 4.0) as described previously.^4^ Error bars show the 95% CI around the mean for each sample. Statistically significant differences occur when the mean of one sample does not lie within the 95% CI of the sample being compared. Fifty cells of each sample were analyzed from each of two biological replicates, giving sample sizes of 100 for each data set.

### Gel electrophoresis

One-dimensional SDS-PAGE was carried out in a conventional way with 12.5% polyacrylamide gels. Two-dimensional gel electrophoresis was carried out by commercial instructions with ampholines (p*I* 4.0–6.5) in the first dimension and a low-Bis 15% acrylamide gel in the second dimension.

### RT-PCR

Samples (80 ml) of predatory cultures from days 1, 3, and 5 described above were treated with 20 ml of 5% phenol in ethanol [final 1% and 19% (v/v)] at 4 °C for 1 h. These were pelleted and stored at − 80 °C, and RNA was prepared by a modification of the Promega SV total RNA isolation kit described elsewhere.^11^ A control of *E. coli* DFB225 only was included. Each RNA sample was diluted to 15 ng/μl as measured by a nanodrop spectrophotometer. RT-PCR was performed using the Qiagen® One-Step RT-PCR kit as described previously^11^ with the following conditions: one cycle of 50 °C for 30 min, 95 °C for 15 min, then 25 cycles of 94 °C for 1 min, 48 °C for 1 min, 72 °C for 2 min followed by one cycle of 72 °C for 10 min then 4 °C hold. Twenty-five to 30 cycles of amplification were used, depending on primer pair, so that the PCR did not go to extinction, allowing a semiquantitative view of mRNA levels at a particular time point. Controls for DNA contamination and cross-reaction with prey were carried out as previously described.^13^

## Figures and Tables

**Table 1:** Comparison of molecular size and p*I*s of multiple flagellins

	Molecular size (aa)	p*I*	Identity *versus* FliC2 (%)
FliC1	274	4.96	55.3
FliC2	277	5.05	100^a^
FliC3	282	4.89	58.4
FliC4	277	5.08	62.0
FliC5	277	5.09	70.4
FliC6	277	5.36	68.5

aa, amino acids.

a FliC2 was used as a model for comparison.
